# Leadless Pacemaker Implantation in Patients With a Prior Conventional Pacing System

**DOI:** 10.1016/j.cjco.2023.12.008

**Published:** 2023-12-15

**Authors:** Julius Jelisejevas, François Regoli, Daniel Hofer, Giulio Conte, Tardu Oezkartal, Ardan M. Saguner, Maria Luce Caputo, Lorenzo Grazioli, Jan Steffel, Angelo Auricchio, Alexander Breitenstein

**Affiliations:** a Department of Cardiology, University Hospital Zurich, Zurich, Switzerland; b Fondazione Cardiocentro Ticino, Lugano, Switzerland; c Ospedale Regionale di Bellinzona e Valli, Bellinzona, Switzerland

## Abstract

**Background:**

Leadless pacing has been established as an alternative approach to transvenous devices for selected patients. Often, leadless pacemaker (LP) implantation is a de novo procedure, but in an increasing number of patients, an LP is used after previous implantation of a conventional pacing system (CPS).

**Methods:**

A retrospective analysis was conducted of the efficacy and safety of LP implantation in the context of a previously implanted CPS, from 2 large Swiss centres.

**Results:**

A total of 257 consecutive patients undergoing LP implantation were included. They were divided into 2 groups: group 1 consisted of 233 patients who did not have a previous CPS, and group 2 consisted of 24 patients with an in situ CPS. In group 2, a total of 20 patients (83%) required transvenous lead extraction due to infection, malfunction, or other reasons. In 3 patients with device-related infection, lead extraction and LP implantation was performed as a single procedure, whereas in the remaining 11 cases, a time window occurred between the 2 procedures (median: 11.5 days; range: 2-186 days). Electrical device parameters at implantation and during follow-up did not differ between the 2 groups (mean: 12.5 ± 9.3 months). Eight major periprocedural complications (3.1%) were encountered (4 pericardial effusions, 3 instances of femoral bleeding, and 1 instance of intra-abdominal bleeding) in the entire cohort within a 30-day period. No complications occurred in the group with a previous device. No infections were registered, even when complete extraction of an infected CPS was performed prior to LP implantation.

**Conclusions:**

Implantation of an LP in patients with a prior CPS (with or without extraction of the previous system) was effective and safe in our population of patients.

## RÉsumÉ

**Contexte:**

La stimulation cardiaque sans fil a été établie comme une solution de substitution aux dispositifs transveineux chez certains patients. L’implantation d’un stimulateur cardiaque sans fil est souvent une intervention *de novo*, mais chez un nombre croissant de patients, ce type de stimulateur est utilisé après l’implantation d’un stimulateur classique.

**Méthodologie:**

Une analyse rétrospective de l’efficacité et de l’innocuité de la stimulation cardiaque sans fil dans le contexte de l’implantation d’un stimulateur classique a été réalisée dans deux grands centres suisses.

**Résultats:**

Un total de 257 patients consécutifs ayant subi l’implantation d’un stimulateur cardiaque sans fil ont été inclus. Les patients étaient répartis dans deux groupes; le groupe 1 était composé de 233 patients non porteurs d’un stimulateur classique, et le groupe 2, de 24 patients porteurs d’un stimulateur classique *in situ*. Dans le groupe 2, 20 patients au total (83 %) ont eu besoin d’une extraction de la sonde transveineuse en raison d’une infection, d’un défaut de fonctionnement, ou pour d’autres motifs. Chez 3 patients présentant une infection liée au stimulateur, l’extraction de la sonde et l’implantation d’un stimulateur cardiaque sans fil ont été réalisées simultanément, tandis que dans les 11 autres cas, il s’est écoulé un temps médian de 11,5 jours entre les deux interventions (min.-max. : 2-186 jours). Les paramètres relatifs au dispositif électrique au moment de l’implantation et pendant le suivi n’étaient pas différents entre les deux groupes (moyenne : 12,5 ± 9,3 mois). Huit complications périopératoires importantes (3,1 %) sont survenues (4 cas d’épanchement péricardique, 3 cas d’hémorragie fémorale et 1 cas d’hémorragie intra-abdominale) dans l’ensemble de la cohorte au cours d’une période de 30 jours. Aucune complication ne s’est produite dans le groupe de patients porteurs d’un stimulateur classique. On n’a enregistré aucun cas d’infection, même lorsque l’extraction complète du stimulateur classique infecté a été effectuée avant l’implantation du stimulateur cardiaque sans fil.

**Conclusions:**

L’implantation d’un stimulateur cardiaque sans fil chez les patients porteurs d’un stimulateur classique (avec ou sans extraction de ce stimulateur) était une intervention efficace et sûre dans cette population de patients.

Permanent pacing, in the absence of reversible causes, has been the main treatment option for bradyarrhythmias since its introduction over 6 decades ago.^1^^,^^2^ Depending on the population studied, the risk of long-term complications with a transvenous device is in the range of 10%-20%, due to device- and lead-associated issues, as well as infection.^3^^,^^4^ These complications are associated with significant morbidity and high mortality rates.^5^^,^^6^ In selected cases, patients need to undergo lead extraction to treat a dysfunctional lead or an infection. In regard to device reimplantation after extraction, the timing and the type of device depend on the reason for the extraction (infection vs non-infection-related issues) and the underlying heart disease.

In patients with relevant bradycardias, the reimplantation of an LP (Micra Transcatheter Pacing System [Micra TPS], Medtronic, Minneapolis, MN),^7^ which carries a lower risk of potential complications, compared to conventional transvenous devices, can be discussed. Indeed, large nonrandomized studies have demonstrated the efficacy and safety of this type of device.^8^^,^^9^ In addition, previously published case series have shown that implantation of an LP performed at the same time as lead extraction in patients suffering from local device infection is safe.^10^

We herein report our experience at 2 large Swiss tertiary centres (University Heart Centre Zurich, Zurich, and Cardiocentro Ticino, Lugano) of patients with a prior conventional pacing system (CPS) who, in the majority of cases, underwent lead extraction, followed by implantation of an LP, either during follow-up or concomitant with the lea—extraction procedure.

## Materials and Methods

### Study population

In this retrospective 2-centre analysis, 257 consecutive patients in a need of single-chamber pacing undergoing LP implantation at 2 high-volume centres in Switzerland (University Hospital Zurich, Zurich, and Cardiocentro Ticino Institute, Lugano) between June 2015 and March 2021 were included. They were divided into 2 groups, as follows: group 1 had 233 patients who did not have a history of having a CPS; group 2 had 24 patients who had a previously implanted CPS for conduction disorders. This study was designed to describe the indications, efficacy, and safety, as well as outcomes, of LP implantation in patients with a previously implanted CPS. The study was approved by the local ethics committee (KEK-ZH-Nr: 2020-00811). All enrolled patients had a clinical indication for a single-chamber pacemaker and provided written informed consent to undergo the procedure.

### Micra TPS implantation

The Micra TPS pacemaker was implanted according to the manufacturer’s recommendation and as previously described.^11^ All procedures were performed in the catheterization laboratory with local anesthesia, and conscious sedation if deemed necessary. Briefly, after gaining femoral venous access (optional ultrasound-guided puncture at the discretion of the operator), a super-stiff wire was advanced into the superior vena cava. After predilatation of the access site, the Micra TPS introducer sheath was advanced into the right atrium. Through this access, the Micra TPS delivery tool, together with the device, was advanced via a 27-F external diameter introducer and positioned into the right atrium. After crossing the tricuspid valve, the device was placed into the septal wall of the right ventricle. When adequate fixation of the device tines by the pull-and-hold test was confirmed, the electrical parameters were tested. If these were within the acceptable range, the tether was cut and slowly pulled out. An intravenous bolus of heparin (between 3000 and 5000 IU, at the discretion of the operator) was routinely administered after insertion of the 27-F external introducer. Hemostasis was assured via a modified figure-of-eight stitch or a Perclose ProGlide device (Abbott, Abbott Park, IL), in all cases.

### Definition of major periprocedural complications

Major procedure-related complications were defined according to previous publications and included death within 30 days as a result of device implantation, and complications prolonging the hospital stay, requiring a new admission, or resulting in significant disability; these included bleeding, pericardial effusion (with or without need of interventional or surgical treatment), permanent loss of device function, device revision within 30 days, infection, device dislodgement, severe damage to the tricuspid valve, and relevant femoral hematoma needing intervention.^8^

### Statistical analysis

Procedural characteristics and outcome data were reviewed and collected from electronic medical records. Statistical analysis was performed using the Jamovi Project (Jamovi Version 1.2; (https://www.jamovi.org). Normality of distribution was assessed for all variables using a Shapiro-Wilk test. Continuous variables were presented as mean ± standard deviation for normally distributed values, or as median (interquartile range [IQR]) for non-normally distributed variables. Categorical variables were presented as counts (%). Comparisons between variables were performed using a Student *t* test, a Mann-Whitney *U* test, a χ^2^ test, or Fisher’s exact test, as appropriate. A 2-tailed *P*-value < 0.05 was considered statistically significant.

## Results

### Study population and baseline characteristics

In this retrospective 2-centre analysis, 257 consecutive patients undergoing LP implantation were included. They were divided into 2 groups, as follows: group 1 had 233 patients who were diagnosed with new-onset bradyarrhythmia in need of device therapy without a history of having a CPS; and group 2 had 24 patients with a previously implanted CPS requiring a new device. Characteristics of the general study population and pacing indications are shown in detail in Table 1. Mean age at implantation was 81.1 ± 8.7 years, and 65% of the population were male. Ischemic heart disease was present as the underlying etiology in 37% of the patients; average left ventricular ejection fraction was 55.5% ± 10%. Seven patients had advanced renal failure, one was on dialysis, and one had a prior CPS implanted. The population with a prior device did not differ in regard to the baseline characteristics, except for the presence of diabetes mellitus, which was significantly less common in this group (4% vs 21%, *P* = 0.047). The main indication for an LP implantation in the control group was slow conducted atrial fibrillation (n = 89; 38%), whereas it was atrial fibrillation or sinus rhythm with complete atrioventricular block in the group with a previous pacemaker system (n = 6 for each; 25%). Characteristics and LP implantation indications in the patient population with a previously implanted device are summarized in Table 2**.** A majority of the patients with a previous CPS underwent lead extraction (n = 20; 83%). This was the case for those with a device infection (n = 14), as well as in 6 patients for whom lead extraction was performed for non-infection-related reasons. In 4 non-infected cases, an LP was implanted, and the old pacing system was left in place. This group included 2 patients with epicardial leads, and 2 patients with a prior implanted dual-chamber pacing system with a fractured right ventricular lead.

**Table 1:** Baseline characteristics and pacing indication of the patient population

Characteristic	All patients (n = 257)	Previous pacemaker (n = 24)	LP only (n = 233)	*P*
Age, y	81.1 ± 8.7	82.7 ± 9.7	81.0 ± 8.6	0.441
Male	167(65)	16 (67)	151 (59)	0.856
LVEF, %	55.5 ± 10.0	54.3 ± 7.4	55.6 ± 10.3	0.434
Underlying heart disease				
Coronary artery disease	94 (37)	8(33)	86 (37)	0.729
Valvular disease	72 (28)	8(33)	64 (27)	0.542
Comorbidities				
Chronic renal failure	141 (55)	17 (71)	124 (53)	0.099
Hemodialysis	7 (2.7)	1 (4.2)	6 (2.6)	0.648
Peripheral artery disease	24 (17)	4 (22)	33 (14)	0.823
COPD	35 (14)	2 (8)	33 (14)	0.428
Diabetes mellitus	50 (19)	1 (4)	49 (21)	0.047
Prior stroke	32 (12)	0 (0)	32 (14)	0.052
Prior pulmonary embolism	7 (3)	0 (0)	7 (3)	0.380
Cancer	40 (16)	5 (21)	35 (15)	0.455
Prior pacemaker	24 (9)	24 (100)	0 (0)	< 0.001
Cardiogenic shock	10 (4)	0 (0)	10 (4)	0.301
AA				
Slow AA	92 (36)	3 (13)	89 (38)	0.012
AA and complete AV block	32 (12)	6 (25)	26 (11)	0.051
Tachy-Brady syndrome	42 (16)	3 (13)	39 (17)	0.593
SR				
Postconversion pause	11 (4)	2 (8)	9 (4)	0.303
Sinus node dysfunction	18 (7)	2 (8)	16 (7)	0.789
SR with complete AV block	42 (16)	6 (25)	36 (15)	0.228
Other causes∗	20 (8)	20 (8)	18 (8)	0.916

Values are presented as mean ± standard deviation, or n (%), unless otherwise indicated.

AA, atrial arrhythmia; AV, atrioventricular; COPD, chronic obstructive pulmonary disease; LP, leadless pacemaker; LVEF, left ventricular ejection fraction; SR, sinus rhythm.

∗ Other causes (left bundle branch block post-transcatheter aortic valve replacement; cardioinhibitory response; syncope prevention, right bundle branch block, and left anterior hemiblock).

**Table 2:** Characteristics and indications for leadless pacemaker (LP) implantation specific to a patient population with a previous conventional pacing system

Characteristic	Value, n (%)
Primary conventional pacemaker	24 (100)
VVI	5 (21)
DDD	17 (71)
CRT	0 (0)
Epicardial system	2 (8)
Indications for LP implantation	14 (58)
Indication for extraction	
Pacemaker-associated infection∗	3 (13)
System malfunction†	3 (13)
Other‡	4 (16)
Not extracted§	

CRT, cardiac resynchronization therapy; DDD, dual-chamber; VVI, single-chamber.

∗ Includes pocket infection, lead infection, or both.

† Fractured right ventricular lead.

‡ Severe tricuspid valve regurgitation in 2 patients, and perforation of right ventricle electrode in 1 patient.

§ Two epicardial systems and 2 DDD systems (fractured right ventricular lead).

### Comparison of procedure characteristics and parameters between the groups

**Figure 1:**
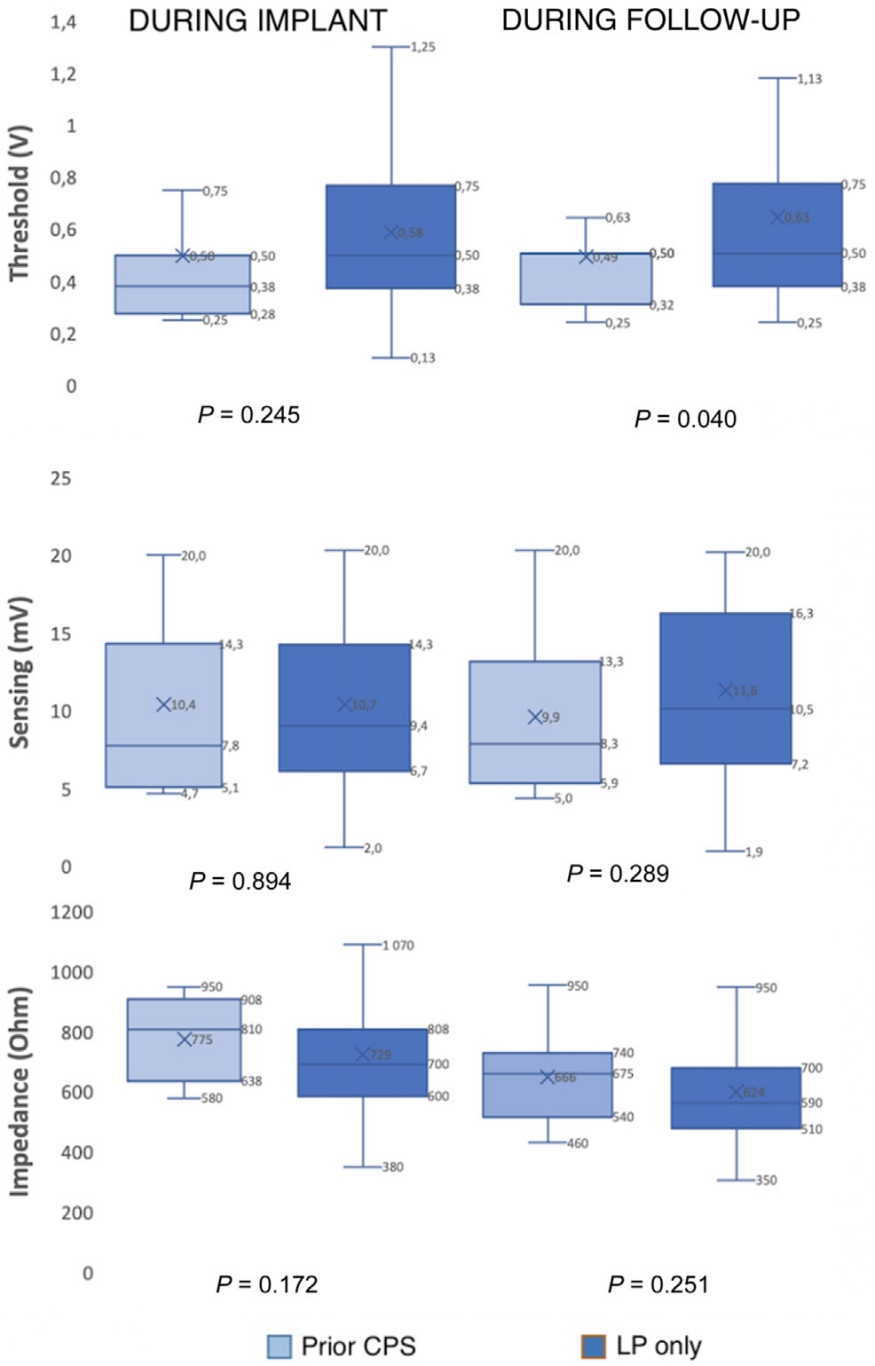
Device parameters for the patient population with a previous conventional pacemaker system (n = 24) vs the leadless pacing–only group at time of implantation and last follow-up. CPS, conventional pacing system; LP, leadless pacemaker.

In the patient population with a prior CPS, the average time of procedure (56.97 ± 31.48 minutes vs 48.24 ± 19.11 minutes; *P* = 0.205), as well as radiation exposure time (10.56 ± 12.76 minutes vs 7.50 ± 7.05 minutes; *P* = 0.266) were numerically longer but without a statistically significant difference between the 2 groups. Although ventricular sensing and device impedance were similar between the 2 groups, a statistically significant, and hence clinically not meaningful difference, in the pacing threshold (0.49 ± 0.22 vs 0.63 ± 0.42 V @ 0.24 ms; *P* = 0.04) during the last follow-up was noted between the 2 groups (Fig. 1). The final LP position in the mid-septal area of the right ventricle was 62.5% (15 of 24) in patients with a prior device vs 58.8% (137 of 233) in the other group (*P* = 0.718).

### Device parameters and characteristics

The final LP position did not differ between the 2 groups and was the mid-septal area of the right ventricle in the majority of the cases (n = 137, 58.8% in the control population; n = 15, 62.5% in the population with a previous device, *P* = not significant). The average threshold was 0.57 ± 0.32 V @ 0.24 ms with sensing of 10.6. ± 4.95 mV and an impedance of 731 ± 165 Ohm for the entire population, without any differences reported between the 2 groups (Fig. 1). To achieve more-optimal electrical parameters in the cohort of patients with a prior CPS and after transvenous lead extraction, the LP device was implanted in a different location from that of the prior transvenous lead position in the right ventricle.

### Procedure characteristics of the patient group with a prior conventional pacemaker system

A total of 24 patients had a prior implanted CPS, in 83% of cases (20 of 24) requiring complete extraction of the former system due to infection, malfunction, or other reasons as summarized in Table 2. Pacemaker-related infection was present in 58% of patients (14 of 24), including either a clinically proven or suspected local infection of the pocket or systemic endocarditis involving device leads. In 3 patients (12.5%) with pacemaker-related infection, the LP was implanted as a single procedure during lead extraction of the conventional system. In the majority of cases with related infection (11 of 14 patients; 78.6%), a time window between complete system extraction and implantation of the LP was used that was a median of 11.5 days (range: 1-186 days). In the 4 non-infection-related instances, an LP was implanted but the preimplanted system was not extracted. In the cases without infection, when transvenous lead extraction was performed, all LP implantations were performed as a single procedure.

### Major periprocedural and 30-day complication rate

Eight major peri-interventional complications (3.1%) occurred during the first 30 days (Table 3). Four pericardial effusions (Pes) occurred, of which 1 was managed conservatively, and 3 required a pericardiocentesis, but none of the patients required surgical drainage, and all 4 recovered without further sequelae. Three PEs were related to LP implantation itself, whereas in a more detailed review, 1 PE that required pericardiocentesis was found to have resulted from the previously implanted and perforated temporary pacing wire, after transcatheter aortic valve replacement followed by complete heart block. The latter was removed during the LP implantation, resulting in a PE. Conservatively managed PE was small in area and required multiple transthoracic echocardiograms, which confirmed spontaneous resolution of the PE. Relevant femoral bleeding was encountered in 3 patients (1 had arterial bleeding of the femoral artery with subsequent covered stenting, and 2 had venous bleeding, which was successfully managed conservatively in both cases). All complications occurred in the de novo cohort. In the 30-day postinterventional period, 1 death occurred, which was not related to LP implantation.

**Table 3:** Major complications in 30-day postoperative period

Characteristics	All patients (n = 257)	Previous pacemaker (n = 24)	Leadless pacing only (n = 233)
Total major complications	8 (100)	0 (0)	8 (100)
Pericardial effusion	4 (50)	0 (0)	4 (50)
Femoral bleeding	3 (38)	0 (0)	3 (38)
Intra-abdominal bleeding∗	1 (13)	0 (0)	1 (13)
Device dislodgement	0 (0)	0 (0)	0 (0)
Device infection	0 (0)	0 (0)	0 (0)

Values are n (%). Because of small numbers, *P* values could not be calculated reliably.

∗ Treated conservatively.

### Outcomes during the follow-up

Pacing threshold, sensing, and impedance were similar between the 2 groups and were within the target range at implantation and at last follow-up for both groups. A total of 22 deaths (8.6%) occurred during follow-up at a median of 334 days (range: 10-1094 days), and none of these deaths were related to the procedure or the device itself.

## Discussion

Leadless pacing is emerging as an alternative option in various settings and may be preferred in older and frail patient populations; it also presents a viable option for patients following transcatheter aortic valve replacement.^12^^,^^13^ Reassuring data show that LP implantation after transvenous lead extraction can be a suitable option in selected patients and is reasonably safe and effective.^14^ In addition to these data, our study provides reassuring data in the patient population with a prior CPS following LP implantation, in relation to indications, procedural characteristics, and outcomes of this patient cohort, compared with the control population. Our main observations are as follows:

1. LP implantation in patients with prior pacemaker systems is safe and effective, showing similar values for pacing threshold, sensing, and impedance at implantation and during follow-up.
2. Procedure and fluoroscopy times are not significantly different between the 2 groups.
3 Procedural outcomes do not differ between the patients with a prior CPS and those in the de novo cohort.
4. The main indication for LP implantation in patients with a prior CPS was device-associated infection requiring total extraction of the former system with a high risk of reinfection.
5. No LP infections have occurred in our group of patients with a prior CPS, even in patients with extracted transvenous leads secondary to device-related infection, independently of whether the strategy of having a time window between the 2 procedures was used, and lead extraction was performed with LP implantation as a single procedure.
6. We report no interference between the 2 systems when the LP was implanted and the CPS was left in situ.
7. Routine utilization of ultrasound-guided puncture should be implemented to potentially mitigate the risk of vascular complications.

### General observations in our patient population

The success rate of LP implantation in the total population was 98.8%, and in the patient population with a prior CPS, it was 100%, which correlates with the data from the Investigational Device Exemption (IDE) Registry (99.2%) and the Post-Market Registry (PAR; 99.1%). Pacing threshold, sensing, and impedance were similar between the 2 groups and were within the target range at implantation and at last follow-up for both groups. The rate of major periprocedural complications in our cohort of consecutive patients undergoing Micra TPS implantation was low, at 3.1%, which is similar to that in other large registries (2.9% in the IDE registry; 1.9% in the PAR at 30 days^9^^,^^15^). Three vascular complications, including one case of arterial bleeding, occurred in our patient cohort. No complications occurred in the patient population who had a previous CPS requiring transvenous lead extraction, or in whom the leads were not extracted. Available data do show that left-sided femoral access can be used successfully and is as safe and effective as a right-sided approach; these data have been previously described and indicate that performing LP implantation during same procedure as lead extraction may be advantageous.^16^

### Leadless pacing after lead extraction in association with device-related infection

An LP is known to reduce the risk of device-related infection, making it a preferred option in cases in which previous infection required a complete extraction of the previous system, as shown previously.^17^^,^^18^ In a patient cohort with a previous device, in the majority of cases, leadless pacing was prioritized due to previous device-related infection, in accordance with current European Society of Cardiology guidelines and suggests that an LP should be considered as an alternative to transvenous pacemakers when the risk of device-pocket infection is particularly high, such as in cases of previous infection and patients on hemodialysis (Class of Recommendation: IIa, Level of Evidence: B).^19^ In the rest of the cases, leadless pacing was chosen due to the advanced age in this frail patient population (Class of Recommendation: IIb, Level of Evidence: C).

We report no device-related infections in our cohort, which represents the combined data from the pivotal IDE Registry study and the PAR, and reports of cases of infection with an LP are very rare. More importantly, no Micra-TPS device-related infections requiring complete extraction of the conventional pacemaker system and a subsequent LP implantation were reported in our subgroup of patients, including in the patient population on hemodialysis. Important factors of leadless pacing contributing to the lack of infection are the absence of a generator pocket, the smaller surface area of the LP, and the turbulence of the blood flow surrounding the Micra TPS device, compared to the laminar, slow flow in the area of the venous system. Taking into consideration that the incidence of device infections increases in frail and older patient populations with repetitive replacements of a pulse generator, our data are highly clinically relevant and confirm that leadless pacing is a safe and reliable option in this patient population.^20^, ^21^, ^22^ After the introduction of the VDD Micra system ("Micra AV", Medtronic, Minneapolis, MN), which offers atrioventricular-synchronized ventricular pacing, even patients in sinus rhythm may now benefit from leadless pacing that avoids pacemaker syndrome and pacemaker-related infection and reinfection.^23^ We report no interactions between the systems either when an LP is implanted and the prior CPS stays in place, or when a time window is planned for the extraction. The data are still limited in these cases regarding continued use of a pulse generator that has not yet reached its end-of-life. According to our findings, if leadless pacing is preferred, it will be efficient and safe, and device performance will be good in patients with a prior device, with or without extraction of the old system.

## Study Limitations

As this is a retrospective, nonrandomized study from 2 experienced centres in Switzerland, the findings may not be representative of other healthcare settings. Larger studies are needed to help establish stronger recommendations for use of an LP for patients who have a prior implanted conventional system and device-related infection.

## Conclusion

LP implantation is safe and effective in patients with a prior CPS, even if total explantation of the former system is needed. In such cases, the implantation success rate is excellent, and the device parameters remain stable during follow-up. The main indication for LP implantation in this group was device-associated infection requiring extraction of the former system to avoid reinfection. We report no interference between the LP and a prior CPS if the latter is left in place. No LP infections occurred in our group of patients with a prior CPS.
